# How diverse can rare species be on the margins of genera distribution?

**DOI:** 10.1093/aobpla/plz037

**Published:** 2019-07-09

**Authors:** Alice Backes, Geraldo Mäder, Caroline Turchetto, Ana Lúcia Segatto, Jeferson N Fregonezi, Sandro L Bonatto, Loreta B Freitas

**Affiliations:** 1 Laboratory of Molecular Evolution, Department of Genetics, Universidade Federal do Rio Grande do Sul, Porto Alegre, Rio Grande do Sul, Brazil; 2 Escola de Ciências, Pontifícia Universidade Católica do Rio Grande do Sul, Porto Alegre, Rio Grande do Sul, Brazil

**Keywords:** Atlantic Forest, highlands, nuclear diversity, open-field species, plastid variability, Pleistocene, rare species, Solanaceae

## Abstract

Different genetic patterns have been demonstrated for narrowly distributed taxa, many of them linking rarity to evolutionary history. Quite a few species in young genera are endemics and have several populations that present low variability, sometimes attributed to geographical isolation or dispersion processes. Assessing the genetic diversity and structure of such species may be important for protecting them and understanding their diversification history. In this study, we used microsatellite markers and plastid sequences to characterize the levels of genetic variation and population structure of two endemic and restricted species that grow in isolated areas on the margin of the distribution of their respective genera. Plastid and nuclear diversities were very low and weakly structured in their populations. Evolutionary scenarios for both species are compatible with open-field expansions during the Pleistocene interglacial periods and genetic variability supports founder effects to explain diversification. At present, both species are suffering from habitat loss and changes in the environment can lead these species towards extinction.

## Teaser

In our study, we used microsatellite markers and plastid sequences to characterize the levels of genetic variation and population structure of two endemic and restricted species (*Petunia mantiqueirensis* and *Calibrachoa elegans*) that grow in isolated areas on the margin of the distribution of their respective genera. Plastid and nuclear diversities were low and weakly structured in their populations. Evolutionary scenarios for both species are compatible with open-field expansions during the Pleistocene interglacial periods and genetic variability supports founder effects to explain diversification. Both species are suffering from habitat loss and changes in the environment can lead these species towards extinction.

## Introduction

Many rare or narrowly distributed species present low levels of genetic diversity (Gitzendanner and Soltis 2000). Additionally, there is a positive association between geographical range size and genetic diversity (Ellstrand and Elam 1993; Hamrick and Godt 1996), especially considering the limits of genera distribution. Species that inhabit the border of the distribution of their respective groups usually have smaller and more fragmented populations and, if there is no immigration between theirs and central populations, they can have fixed locally adapted alleles (Bridle and Vines 2006).

Ecological and evolutionary processes define the distribution of species across space and time. The climatic changes that occurred during the Quaternary impacted the distribution and genetic diversity of most species through cycles of range contraction and expansion in the northern and southern hemispheres (Hewitt 2000; Behling 2002; Beheregaray 2008), and, most recently, acquired heightened relevance in relation to current global warming (Lavergne *et al.* 2010). Climate change alters local conditions and can cause range expansion and contraction affecting the species distribution and this range dynamics may drive the processes of diversification across time and space (Dawson *et al.* 2011). Geographic range expansion during interglacial periods and the associated founding effects are expected to reduce allelic richness and increase homozygosity (Pfeifer *et al.* 2009) compared to species at the centre of genus distribution.

**Figure 1.**
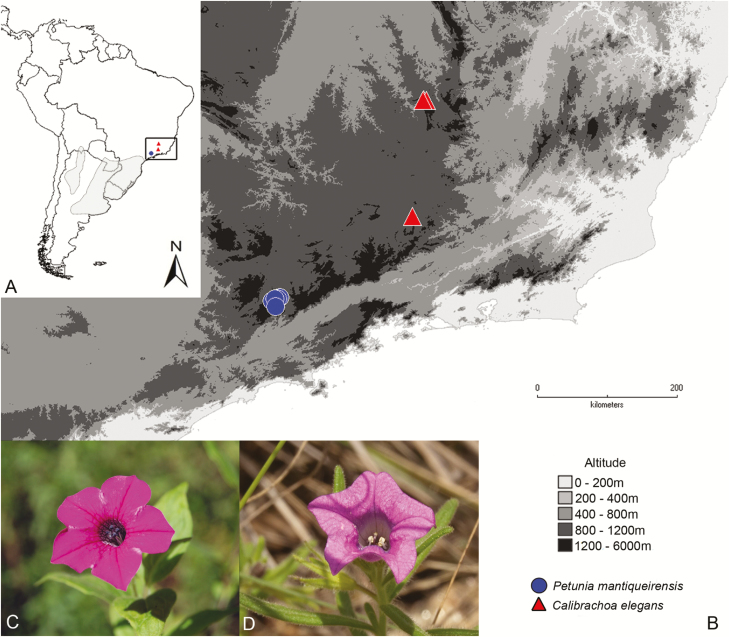
(A) Rectangle area corresponds to the geographical distribution of *Petunia mantiqueirensis* and *Calibrachoa elegans*. The grey area corresponds to the distribution of other species from both genera; (B) collection sites; (C) *Petunia mantiqueirensis* flower; and (D) *Calibrachoa elegans* flower (Photos J. R. Stehmann, UFMG).

Some studies have focused on the evolutionary history of species adapted to open areas interspersed among regions of higher altitude in the southern section of the Atlantic Forest (Behling 2002; Safford 2007). The south-eastern Brazilian highlands, which are located in the subtropical region of South America, consist of a mosaic of grasslands and Atlantic mixed forest. These formations occur between latitudes 18° and 24°S at elevations greater than 1400 m in small isolated areas (Fig. 1A and B). The relationship between grassland and forest during the late Quaternary in this region was documented through pollen records and probably resembles the general pattern of shifts in vegetation range that took place throughout the Pleistocene period (Ledru 1993; Ledru *et al.* 2005).

Species of *Petunia* and *Calibrachoa* share several evolutionary constraints and biological characteristics, many times occurring sympatrically, side by side. Based on a proposed origin in open fields (Olmstead *et al.* 2008) at ca. 8.5 Mya (Särkinen *et al.* 2013), these genera were strongly influenced by the Pleistocene glacial and interglacial cycles (between ca. 2.8 and 1.3 Mya) during their diversification (Lorenz-Lemke *et al.* 2010; Fregonezi *et al.* 2013; Särkinen *et al.* 2013; Barros *et al.* 2015). The ancestor for both has Andean origin (Reck-Kortmann *et al.* 2015) that posteriorly dispersed to the north and colonized the highlands (Reck-Kortmann *et al.* 2014). Both genera include endemic and restricted taxa, frequently associated with specific micro-environments (Stehmann *et al.* 2009; Fregonezi *et al.* 2012).

During the last glaciation, the southern and south-eastern Brazilian highlands were predominantly grasslands. Cold and dry climate conditions allowed the grasslands to expand northwards, where small populations of *Araucaria angustifolia* were restricted to more humid areas along shadowy ravines (Behling *et al.* 2001; Ledru *et al.* 2005). As temperature and humidity increased in the early Holocene, the *Araucaria* forest expanded over the grasslands and only the highest areas in this region during the interglacial periods were refuges for species previously associated with open areas (Behling 2002), with many cold-adapted species still remaining there (Lorenz-Lemke *et al.* 2010). Strong influence of global climate changes during the Pleistocene on the evolutionary processes in plants has been described for several species from the south-eastern region of Brazil [*Hymenaea stigonocarpa* (Ramos *et al.* 2007); *Lychnophora ericoides*, Collevatti *et al.* (2009); *Epidendrum fulgens*, Pinheiro *et al.* (2011); *Tabebuia aurea*, Collevatti *et al.* (2015); and *Eugenia uniflora*, Turchetto-Zolet *et al.* (2016)], as well as to species that occur closest to the Atlantic coast, such as *Calibrachoa heterophylla* (Mäder *et al.* 2013), *Recordia reitzii* (Thode *et al.* 2014), *Petunia integrifolia* (Ramos-Fregonezi *et al.* 2015) and *Passiflora contracta* (Cazé *et al.* 2016).

Among the species of *Petunia* and *Calibrachoa*, *P. mantiqueirensis* and *C. elegans*, respectively, have reached the northernmost distribution of their respective genera. Both species are endemic of a very specific habitat at the edge of each genus distribution and are found in small patches with few individuals.

As genetic drift tends to further reduce genetic diversity (Ellegren and Ellegren 2016; Thurman and Barrett 2016), the fixation of alleles because of low effective sizes is a phenomenon that more commonly occurs on the edge of the range of distribution of a species where expansion and contraction events are frequent (Eckert *et al.* 2008; Excoffier *et al.* 2009; Angert 2014; Hargreaves and Eckert 2014). As such, our hypothesis was that *P. mantiqueirensis* and *C. elegans* present lower genetic variability compared to their congeners, which is related to their rarity and limited occurrence at the edge of their respective genera distribution owing to a pronounced founder effect.

Within this context, the main goal of this study was to assess the genetic diversity of these two rare and endemic species using plastid DNA sequences and nuclear microsatellite markers. We discuss their genetic diversity, comparing these with congeneric species aiming to provide elementary information about the consequences of the expansion of these genera’s distribution and, indirectly, to contribute to the establishment of conservation strategies for these rare and endangered species.

## Methods

### Plant material

*Petunia mantiqueirensis* (Fig. 1C) is endemic to high altitude regions in Serra da Mantiqueira, Minas Gerais Brazilian state (elevations > 1300 m), specifically in mixed ombrophilous forest (*Araucaria* moist forests), which is the northernmost distribution of the *Petunia* genus. This species is annual and herbaceous, with bee-pollinated flowers displaying a purple and short corolla tube (Stehmann *et al.* 2009). The area where the species is found is fragmented because of urbanization and intense agricultural activity (Kamino *et al.* 2012).

*Calibrachoa elegans* (Fig. 1D) is a micro-endemic, annual and herbaceous species from the Iron Quadrangle (Minas Gerais, south-eastern Brazil) of which only four occurrence sites are known. The species has the northernmost distribution within the genus and occurs in a mountainous landscape (elevation > 1000 m), strongly corrugated, and dominated by grassland, popularly called ferruginous field from ‘nodular canga’ (Fregonezi *et al.* 2012). The species has unique adaptations to the climate of the region, which presents a rigorous rainfall regime, with 4 months of dry season. It is self-incompatible and bee-pollinated (Stehmann and Semir 2001).

These two species are classified as federally endangered according the Brazilian Red List (CNCFlora, available at http://cncflora.jbrj.gov.br/portal). We sampled all known populations and adult individuals found in the same flowering season [for *P. mantiqueirensis*, seven sites (Mant1 to Mant7) totalling 38 individuals, and for *C. elegans*, 81 individuals from four sites (Eleg1 to Eleg4)]. Vouchers were deposited at the BHCB herbarium (Universidade Federal de Minas Gerais, Belo Horizonte, Minas Gerais, Brazil—UFMG) and the geographical coordinates for all collection sites were obtained with a global positioning system (GPS) unit **[see****Supporting Information—Table S1****]**. We collected three or four young leaves from each adult individual of both species while seeking to minimize damage to the plants; we dried leaves in silica gel and then pulverized them in liquid nitrogen for DNA extraction.

### DNA extraction, amplification, sequencing and genotyping

The genomic DNA was extracted from the powdered leaves with a cetyl trimethyl ammonium bromide (CTAB) protocol (Roy *et al.* 1992) and the quality and quantity was evaluated by measuring absorbance at 260 and 280 nm on a Nanodrop spectrophotometer (Thermo Scientific Corp., San Jose, CA, USA).

We estimated the genetic diversity based on the plastid sequences (cpDNA) of intergenic spacers, *trnH-psbA* and *trnS-trnG*, were amplified and sequenced for 17 individuals of *P. mantiqueirensis* and 81 individuals of *C. elegans* using universal primers (Hamilton 1999; Sang *et al.* 1997, respectively). The PCR reactions were performed in 25-µL final volumes consisting of 1 U *Taq* polymerase (Invitrogen, Carlsbad, CA, USA), 1× buffer (Invitrogen), 0.2 mM each dNTP, 2 mM MgCl_2_, 0.2 µM of each primer and 10 ng of genomic DNA. The amplification conditions for *trnH-psbA* and *trnS-trnG* were conducted as described previously for *Petunia* species (Lorenz-Lemke *et al.* 2006). The PCR products were verified by horizontal electrophoresis in 1 % agarose gel dyed with GelRed™ (Biotium, Inc., Hayward, CA, USA) and purified with polyethylene glycol 20 % (Dunn and Blattner 1987). Sequencing was performed with a Mega BACE 1000 (GE Healthcare Bio Sciences Corp., Piscataway, NY, USA). We also included in our analyses the sequences of 21 individuals of *P. mantiqueirensis* as previously described (Lorenz-Lemke *et al.* 2010).

To evaluate current variability in each population and species, individuals of *P. mantiqueirensis* were genotyped with 12 microsatellite loci markers (SSR) previously developed for *Petunia hybrida* (Bossolini *et al.* 2011) adhering to the protocol described for wild *Petunia* species (Turchetto *et al.* 2015). For *C. elegans*, we analysed 10 microsatellite loci developed for *C. heterophylla* (Silva-Arias *et al.* 2015) and four markers developed for *C. pygmaea* (G. Mäder *et al*., unpubl. data). We amplified SSR regions in a final volume reaction of 10 µL containing ~10 ng of genomic DNA as a template, 200 µM of each dNTP (Invitrogen), 1.7 pmol of fluorescently labelled M13(-21) primer, 3.5 pmol of reverse primer, 0.35 pmol of forward primer with a 5′-M13(-21) tail, 2.0 mM MgCl_2_ (Invitrogen), 0.5 U of Platinum *Taq* DNA polymerase (Invitrogen), and 1× Platinum *Taq* reaction buffer (Invitrogen). The PCR conditions were as follows: an initial denaturation at 94 °C for 3 min; 30–35 cycles of 94 °C for 20 s, 48–58 °C for 45 s and 72 °C for 1 min; and a final extension cycle at 72 °C for 10 min.

The forward primers were FAM-, NED-, HEX-, VIC- and PET-labelled. The amplified product was visualized on a 2.5 % ultra-resolution agarose gel stained with 2 µL 0.001 % GelRed™. The amplified DNA was denatured and size-fractionated employing capillary electrophoresis on an Applied Biosystems (Foster City, CA, USA) with a LIZ (500) molecular size standard (Applied Biosystems). GeneMarker 1.97 software (Softgenetics LLC, State College, PA, USA) was utilized to determine the alleles. Additionally, all alleles were visually verified and scored. Repeat motifs, respective annealing temperatures and fluorescent dyes used in each fragment are presented in **Supporting Information—Table S2**.

### Plastid variability

For each plastid marker, both forward and reverse strands were assembled using ChromasPro 1.7.5 (Technelysium Pty Ltd, Australia) and haplotype sequences were deposited at GenBank (available at https://www.ncbi.nlm.nih.gov/genbank/) under numbers: *C. elegans*JQ082471.1, JQ082474.1, KM982182.1, KM982262.1; *P. mantiqueirensis*AY772921.1, DQ792340.1. DNA sequences were aligned using MEGA7 software (Kumar *et al.* 2016) with the ClustalW algorithm, and manually edited when necessary. Haplotype (*h*) and nucleotide (π) diversity indexes and the number of variable sites were calculated with Arlequin 3.5.1.2 (Excoffier and Lischer 2010). The two plastid intergenic spacers were concatenated and treated as a single sequence for all analyses. The numbers of variable and informative sites in the manually edited alignment were obtained from Mega7 software. Haplotypes were identified via DNAsp 5.10.01 (Rozas *et al.* 2003).

### Nuclear diversity

For both species, we used Fstat 2.9.3.2 software (Goudet 1995) to evaluate statistics, such as allelic frequencies, the number of alleles per locus (*A*), gene diversity (GD), allelic richness (AR) and inbreeding coefficient (*F*_IS_) for each locus. The frequencies of the null alleles, polymorphic index content (PIC), levels of observed (*H*_o_) and expected (*H*_e_) heterozygosity, and any significant deviations from the Hardy–Weinberg equilibrium (HWE), in order to evaluate the informativeness of the markers, were estimated using Cervus 3.0.3 software (Marshall *et al.* 1998; Kalinowski *et al.* 2007).

We performed locus-by-locus analysis of molecular variance (AMOVA) (Excoffier *et al.* 1992) and the overall *F*_ST_ to evaluate the partition of the genetic variation among populations. The AMOVA analysis was performed in Arlequin employing 10^4^ permutations. We also estimated pairwise *F*_ST_ to evaluate the level of genetic differentiation between populations of both species.

Genetic relationships among populations of each species were also examined by applying the discriminant analysis of principal components (DAPC; Jombart *et al.* 2010) on the SSR data set with the Adegenet package (Jombart 2008) without locational priors included in the analysis.

### Test of alternative demographic scenarios

**Figure 2.**
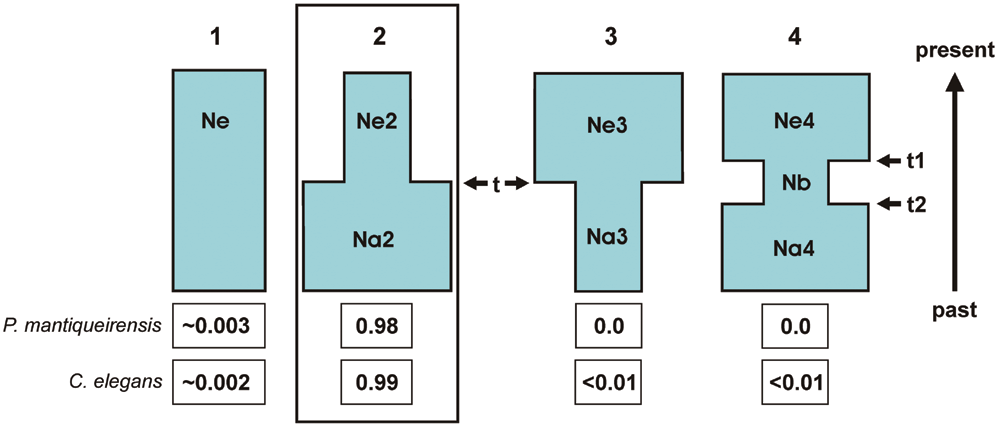
Demographic scenarios for *Petunia mantiqueirensis* and *Calibrachoa elegans*. The four demographic scenarios tested with the DIYABC approach: 1—constant population; 2—bottlenecked population; 3—expanded population; 4—population with a transitory bottleneck. Demographic parameters: Ne—long-term; Ne2, Ne3 and Ne4—current; Na2 and Na4—pre-bottleneck; Na3 and Nb—during bottleneck; *t*, time of single demographic change; *t*_2_ and *t*_1_, beginning and end of bottleneck. Prior values for the parameters are in **Supporting Information—Table S3**. The posterior probability (logistic approach with the 8000 closest simulations) of each scenario is given at the bottom.

An approximate Bayesian computation (ABC) approach (Bertorelle *et al.* 2010) implemented in the program Diyabc 2.1.0 (Cornuet *et al.* 2014) was used to test four plausible colonization scenarios for each of these two species based on previous studies (Stehmann *et al.* 2009; Fregonezi *et al.* 2012; Reck-Kortmann *et al.* 2014) employing microsatellite variation. Scenario 1 is of a long-term constant size population (no bottleneck), scenario 2 consisted of a population that is still experiencing a bottleneck, scenario 3 is a population that expanded recently from a bottleneck and scenario 4 is a population that experienced a transitory bottleneck (Fig. 2). The priors for all demographic parameters were uniformly distributed between specified minimum and maximum values **[see****Supporting Information—Table S3****]**, which were based on the available information surrounding *Petunia* and *Calibrachoa*.

The demographic parameters were: scenario 1: Ne (long-term Ne); scenario 2: Ne2 (current Ne), Na2 (pre-bottleneck Ne); scenario 3: Ne3 (current Ne), Na3 (Ne during bottleneck); *t* (time since demographic change in scenarios 2 and 3); scenario 4: Ne4 (current Ne), Nb (Ne during bottleneck), Na4 (pre-bottleneck Ne), *t*_2_ and *t*_1_ (time since the beginning and end of the bottleneck, respectively). Demographic prior values are presented in **Supporting Information—Table S3**. For population size parameters we used broad priors as we do not have much previous information on them. As priors for the times for the bottleneck scenario (t_2_ and t_1_, scenario 4), we first used as reference the maximum the influence of the Last Glacial Maximum (LGM, ~21 kya) and as the minimum the first record of *Araucaria* forest expansion in the region [~8 kya (Ledru *et al.* 2005), plus or minus 4 kya for uncertainties]. After the first results, we adjusted prior values to the following: extended *t* and *t*_2_ maximum time to 50 kya and reduced time *t* to a minimum of 10 years ago. Mutational parameter priors were set to evolve under the generalized stepwise mutation model (GSM; Estoup *et al.* 2002) with mean mutation rate (μ) uniformly ranging from 10^−4^ to 10^−2^ and the individual locus mutation rate per generation range from 10^−5^ to 10^−2^ (Zhang and Hewitt 2003) with gamma distribution with shape equal to 2. Generation time was set to 1 year. Motif sizes and allele ranges followed the empirical data of each locus **[see****Supporting Information—Table S2****]**. The four single sample summary statistics available were used (mean number of alleles, mean gene diversity, mean allele size and mean M index all across loci). A total of 8 000 000 simulations (2 000 000 for each scenario) were carried out to generate the reference table. The posterior probability of each scenario was assessed employing both logistic regression and direct estimate approaches utilizing 40 000 and 500 best simulations, respectively. For the best scenario, the posterior distribution of the parameters was estimated using *logit* transformation for the 20 000 best simulations. We also used several options in DIYABC to evaluate the confidence in scenario choices, posterior model checking and determination of bias and precision of the parameter estimation. The details of these analyses are described in **Supporting Information—Table S4**.

### Ecological niche modelling

Ecological niche modelling (ENM) was used to compare niche preferences and potential range shifts that the species can suffer from in response to future climatic oscillations. Localities from the two species were obtained from field observations by the authors, SpeciesLink (http://www.splink.org.br) and the Global Biodiversity Information Facility (http://www.gbif.org). After excluding duplicated, false positive records and spatially autocorrelated points, we used information for 13 localities of *P. mantiqueirensis* and 11 of *C. elegans***[see****Supporting Information—Table S5****]**. We employed Maxent 3.4.1 (Phillips *et al.* 2006) to run models for each species varying the feature classes and the regularization multiplier (Galante *et al.* 2018). We used five different feature classes (linear; hinge; linear and quadratic; linear, hinge and quadratic; default) and five values for the regularization multiplier (1–5, with increments of 1) for each species, for a total of 50 models. To evaluate the models, we used ENMTools (Warren *et al.* 2010; Warren and Seifert 2011) and the best model was chosen according the AICc value. The best model indicated was used to project two future conditions, and three past conditions using 3-fold cross-validation. To model future distributions, we used 2050 (average for 2041–60) and 2070 (average for 2061–80) conditions, with the Community Climate System Model (CCSM) and the Representative Concentration Pathways (RCP 6.0). The RCPs are calculated considering a possible range of radioactive forcing values in the year 2100 relative to pre-industrial values, and RCP 6.0 considers a value of +6.0 W m^−2^. To model past distributions, we used Mid-Holocene (~6 kya), Last Glacial Maximum (LGM; ~21 kya) and Last Interglacial (LIG; ~120–140 kya) conditions. Explanatory variables included a set of 19 bioclimatic Raster layers at a 30 arc-second resolution (ca. 1 km^2^ at the Equator) from the WorldClim website, version 1.4 (available at: http://www.worldclim.org/). The Raster package (Hijmans and van Etten 2010) implemented in R software (available at: http://www.R-project.org) was used to calculate Pearson’s correlation between variables. For *P. mantiqueirensis*, four variables (06—Minimum Temperature of Coldest Month; 09—Mean Temperature of Driest Quarter; 15—Precipitation Seasonality; and 17—Precipitation of Driest Quarter) with pairwise Pearson correlation coefficients below 0.7 and with a high percentage of model contribution (in preliminary runs with all variables) were used to reconstruct the taxon distribution. For *C. elegans*, we employed seven variables (02—Mean Diurnal Range; 05—Maximum Temperature of Warmest Month; 06—Minimum Temperature of Coldest Month; 07—Temperature Annual Range; 09—Mean Temperature of Driest Quarter; 15—Precipitation Seasonality; and 16—Precipitation of Wettest Quarter).

## Results

### Plastid diversity

The plastid intergenic spacers, *trnH-psbA* and *trnS-trnG*, for 38 individuals of *P. mantiqueirensis* resulted in a concatenated alignment of 1121 base pairs (bp) long (471 bp corresponding to *trnH-psbA* and 650 bp to *trnS-trnG*). The sequences did not present any variable site, producing a single haplotype. The GC content was 28.1 % in *trnH-psbA* and 30.5 % in *trnS-trnG.* For *C. elegans*, the sequences of 81 individuals produced a concatenated alignment with 1191 bp (445 bp for *trnH-psbA* and 746 bp for *trnS-trnG*), leading to two different haplotypes. The GC content was 27.2 % in *trnH-psbA* and 30.7 % in *trnS-trnG*. Haplotype diversity in *C. elegans* was *h* = 0.0299 ± 0.0287, and nucleotide diversity was π = 0.004 ± 0.014 %. The frequency of each haplotype was CEh1 = 0.98 and CEh2 = 0.02; CEh2 was observed only at one collection site (Eleg1).

### Nuclear diversity and structure

The 38 individuals of *P. mantiqueirensis* were genotyped using 12 SSR loci, being 10 polymorphic and two monomorphic (PM110 and PM195) for this species. All loci were in linkage equilibrium (*P* < 0.001; Bonferroni’s adjusted value for a nominal level of 5 %). The 81 individuals of *C. elegans* were genotyped based on 14 SSR loci, from which five markers were polymorphic and nine monomorphic (CHE12, CHE46, CHE48, CHE114, CHE119, CPY29, CPY31, CPY58 and CPY144). All analyses were conducted based exclusively on polymorphic markers for each species, although the monomorphic loci are suggestive of low genetic variability and high homogeneity in these species as all of them are polymorphic among other *Petunia* and *Calibrachoa* species, respectively.

*Petunia mantiqueirensis* presented a total of 39 alleles, with an average of 3.9 alleles per locus (2 to 7). The mean PIC value was 0.39 (0.05 to 0.75), and considering all loci and gene diversity, it was, on average, 0.36 (0.06 to 0.80). Allele richness was, on average, 3.33 (2.00 to 7.00). Six loci exhibited a significant deviation from HWE across all samples, three of them had a deficit of heterozygotes and the other three displayed an excess of heterozygotes. Tests for HWE indicated no significant *F*_IS_ values for all loci and populations and the mean estimated frequency of null alleles was lower than 0.5 % (Table 1). When we analysed each population as a unit, we observed four loci with a significant deviation from HWE in two populations (Mant1 and Mant3), and three loci presented a deficit of heterozygotes and one an excess of heterozygotes. Four populations presented private alleles (Mant1, Mant3, Mant5 and Mant6) **[see****Supporting Information—Table S6****]**. When we consider the species’ global values we observed no significant HWE deviation or *F*_IS_ values (Table 2).

**Table 1.** Genetic diversity indices based on the microsatellite profile in *Petunia mantiqueirensis. A*, number of alleles per locus; PIC, polymorphic index content; GD, gene diversity; AR, allele richness; *H*_o_, observed heterozygosity; *H*_e_, expected heterozygosity; *F*_IS_, inbreeding coefficient; NUL, frequency of null alleles. *Significant HWE deviation after Bonferroni correction at *P* = 0.05.

Locus	*A*	PIC	GD	AR	*H*_o_	*H*_e_	*F*_IS_	NUL (%)
PM8	4	0.388	0.429	3.912	0.400	0.429	0.068	0.023
PM167	7	0.582	0.619	6.621	0.667	0.619	−0.077	−0.057
PM101	3	0.195	0.213	2.923	0.128*	0.211	0.397	0.232
PM117	3	0.578	0.662	3.000	0.625	0.661	0.055	0.020
PM177	3	0.232	0.251	2.998	0.175*	0.250	0.302	0.159
PM21	2	0.187	0.211	2.000	0.237	0.212	−0.121	−0.058
PM184	5	0.579	0.650	4.969	0.775*	0.651	−0.193	−0.115
PM191	3	0.055	0.057	2.600	0.029*	0.057	0.500	0.410
PM173	7	0.754	0.798	7.000	0.929*	0.800	−0.164	−0.091
PM63	2	0.354	0.462	2.000	0.714*	0.466	−0.545	−0.217
Mean	3.9	0.390	0.363	3.335	0.390	0.363	−0.075	0.031

**Table 2.** Characterization of the 10 microsatellites for *Petunia mantiqueirensis*. All populations values considering pooled populations; *N*, number of alleles; *E*, number of private alleles; *H*_e_, expected heterozygosity; *H*_o_, observed heterozygosity; *F*_IS_, inbreeding coefficient. *Significant HWE deviation after Bonferroni correction at *P* = 0.05; – not estimated.

		Mant1	Mant2	Mant3	Mant4	Mant5	Mant6	Mant7	All populations
*Petunia mantiqueirensis*	*N*	27	21	30	14	23	14	14	39
	*E*	3	–	3	–	1	1	–	–
	*H*_e_	0.492	0.616	0.441	0.625	0.617	0.667	0.667	0.435
	*H*_o_	0.579	0.667	0.524	0.875	0.667	1.000	1.000	0.468
	*F*_IS_	−0.033	−0.130	−0.199	−0.750	−0.109	−1.000	−1.000	−0.075

The AMOVA indicated that the percentage of variation within populations (91 %) was higher than among populations (9 %), and the fixation index (*F*_ST_) over all loci was 0.087 (*P* < 0.05). Five *F*_ST_ pairwise comparisons had significant genetic differentiation, ranging from 0.07 to 0.25 (*P* < 0.05). The Mant3 had significant differentiation from four other populations (Mant1, Mant4, Mant5 and Mant6), as well as Mant4 vs. Mant1 (*F*_ST_ = 0.25) **[see****Supporting Information—Table S7****]**. With the DAPC analysis, the individuals from a same collection site did not preferentially form groups and individuals from Mant4 and Mant7 were the most differentiated **[see****Supporting Information—Fig. S1A****]**.

*Calibrachoa elegans* also presented a total of 39 alleles, with an average of 7.8 alleles per locus (4 to 13) and the PIC values for these markers ranged from 0.47 to 0.84 (average 0.63). The gene diversity was, on average, 0.54 (0.45 to 0.73) and allele richness 5.32 (3.06 to 8.42). All loci exhibited a significant departure from HWE across all samples with heterozygote deficits (mean *H*_o_ = 0.40 and *H*_e_ = 0.68 across all samples). Moreover, three of them had significant and positive *F*_IS_ values, whereas one presented a significant and negative *F*_IS_ value. The estimated frequency of null alleles was lower than 0.5 % in *C. elegans* (Table 3) as in *P. mantiqueirensis* (Table 1). When the analysis was performed per population, we observed that a different number of loci exhibited a significant departure from HWE on populations with a positive and significant inbreeding coefficient (*F*_IS_) **[see****Supporting Information—Table S8****]**. Eleg2 had the highest average of *F*_IS_ with one locus presenting significantly positive values. Furthermore, the populations had different numbers of loci with significant deviations from HWE, and all presented a deficit of heterozygotes at least in one locus. All populations also featured private alleles. Note that Eleg2 was the site with the lowest genetic diversity as measured by number of alleles (*A*) and allele richness (AR) **[see****Supporting Information—Table S8****]**. When we consider the species’ global values (Table 4) we found negative and significant *F*_IS_.

**Table 3.** Genetic diversity indices based on the microsatellite profile in *Calibrachoa elegans. A*, number of alleles per locus; PIC, polymorphic index content; GD, gene diversity; AR, allele richness; *H*_o_, observed heterozygosity; *H*_e_, expected heterozygosity; *F*_IS_, inbreeding coefficient; NUL, frequency of null alleles. *Significant HWE deviation after Bonferroni correction at *P* = 0.05.

Locus	*A*	PIC	GD	AR	*H*_o_	*H*_e_	*F*_IS_	NUL (%)
CHE33	13	0.841	0.729	8.422	0.473*	0.863	0.351*	0.287
CHE34	6	0.471	0.498	4.163	0.390*	0.506	0.305*	0.105
CHE59	4	0.478	0.463	3.061	0.421*	0.565	0.081	0.140
CHE85	10	0.682	0.452	5.981	0.481*	0.724	−0.095*	0.197
CHE 126	6	0.668	0.547	4.983	0.254*	0.721	0.604*	0.488
Mean	7.8	0.628	0.538	5.322	0.404	0.676	0.249	0.243

**Table 4.** Characterization of the five microsatellites for *Calibrachoa elegans.* All populations values considering pooled populations; *N*, number of alleles; *E*, number of private alleles; *H*_e_, expected heterozygosity; *H*_o_, observed heterozygosity; *F*_IS_, inbreeding coefficient. *Significant HWE deviation after Bonferroni correction at *P* = 0.05; – not estimated.

		Eleg1	Eleg2	Eleg3	Eleg4	All populations
*Calibrachoa elegans*	*N*	27	10	28	22	39
	*E*	3	2	8	1	–
	*H*_e_	0.653	0.241	0.653	0.630	0.675
	*H*_o_	0.620	0.051	0.494	0.503	0.404
	*F*_IS_	0.052	0.794	0.251	0.214	0.404*

The AMOVA analysis revealed that the percentage of variation within populations (72 %) was higher than among populations (28 %) and the fixation index (*F*_ST_) over all loci was 0.281. *F*_ST_ pairwise comparisons had significant and strong genetic differentiation in just the Eleg2 compared to the other sites (Eleg2 vs. Eleg1 = 0.53; Eleg2 vs. Eleg3 = 0.55 and Eleg2 vs. Eleg4 = 0.49; *P* < 0.05). With the DAPC, individuals from Eleg2 were completely differentiated from the others, whereas a high superimposition was observed among the other collection sites **[see****Supporting Information—Fig. S1B****]**.

### Demographic scenarios

The comparison of the four demographic scenarios for *P. mantiqueirensis* and *C. elegans* using the ABC approach reflected that the most likely scenario for both species is that in which the population size in the past was much greater than in the present (Fig. 2). Support for this scenario in *C. elegans* was very high according to the logistic approach (above 0.98), and a little smaller with the direct approach, above 0.90 (95 % CI = ~0.65–1.0), and extremely high for *P. mantiqueirensis* above 0.99 and 1.0, respectively. Confidence in the choice of these scenarios were high for the *C. elegans* and very high for *P. mantiqueirensis*, in which the predictive errors were 0.16 and 0.0, respectively **[see****Supporting Information—Table S4****]**. The posterior model checking for scenario 2 in *P. mantiqueirensis* presented a good fit while for *C. elegans* the fit for one of the summary statistics was not good **[see****Supporting Information—Table S4****]**. The posterior density estimates of the demographic parameters for scenario 2 are found in **Supporting Information—Table S4** and Table 5. The bias and error in scenario 2 were very low for all parameters in *C. elegans* while it is higher in *t* for *P. mantiqueirensis***[see****Supporting Information—Table S4****]**. The estimated time since the population reduction presented a wide confidence interval, the model time for *P. mantiqueirensis* was older than for *C. elegans* (~19 and ~11 kya, respectively) and it was not younger than ~5 kya for either (Table 5).

**Table 5.** Posterior parameter estimates based on ABC scenario 2.

	Parameter	Mode	q050	q950
*Petunia mantiqueirensis*	Ne2	534	353	1330
	*t*	2000	901	22 620
	Na2	77 200	33 200	468 000
*Calibrachoa elegans*	Ne2	4880	3180	13 000
	*t*	25 200	9030	47 400
	Na2	477 000	36 700	483 000

### Niche preferences and future adequacy

**Figure 3.**
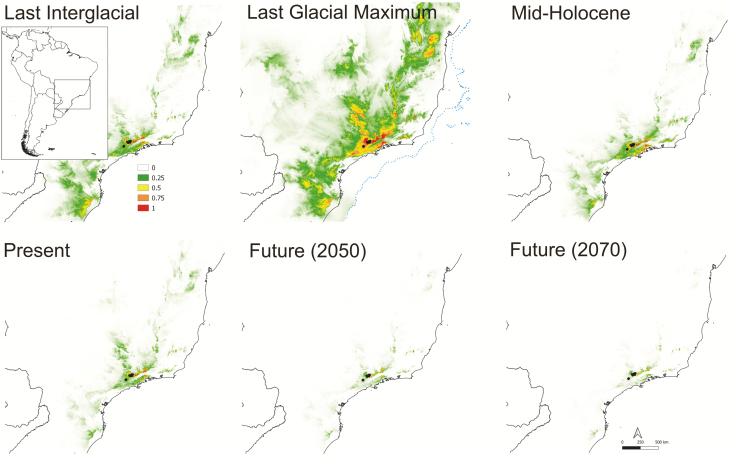
Ecological niche modelling for *Petunia mantiqueirensis* obtained with Maxent. Black dots represent species localities in which the models were based. Colour bars indicate suitability scores represented for *P. mantiqueirensis.* The coast line in the LGM is indicated by a dotted line.

**Figure 4.**
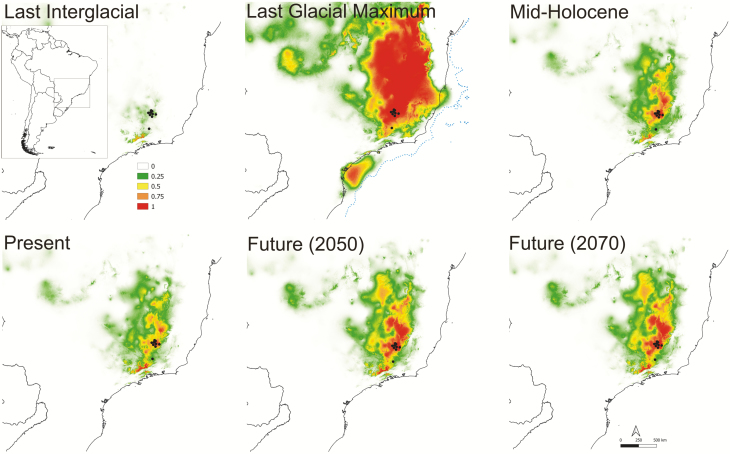
Ecological niche modelling for *Calibrachoa elegans* obtained with Maxent. Black dots represent species localities in which the models were based. Colour bars indicate suitability scores represented for *C. elegans*. The coast line in the LGM is indicated by a dotted line.

Considering the area under the ‘receiver operating characteristic (ROC) curve’ (AUC) (Peterson *et al.* 2008; Elith and Leathwick 2009) all tested models had AUC values above 0.9 and could be considered robust model performance (Fielding and Bell 1997). However, when the AICc was analysed the best model was the one using default feature classes and regularization multiplier equal to one for both species **[see****Supporting Information—Table S9****]**. Precipitation seasonality was the most important ecological variable to model *P. mantiqueirensis* niche adequacy, whereas temperature annual range was the most imperative for *C. elegans*. In the simulated model for *P. mantiqueirensis* at present time (Fig. 3), suitable habitats were indicated slightly further to the north than the current distribution, still in the Serra da Mantiqueira (Minas Gerais, Brazil), and even less pronounced to the west in Serra do Mar (Rio de Janeiro, Brazil). The new suitable habitats indicated for *C. elegans* (Fig. 4) were further to the north and south from the present range still in the canga soils. ENM for future scenarios showed a slight increase in distribution for *C. elegans* and a slight reduction when *P. mantiqueirensis* is considered. When projected to past conditions, the largest distribution was during the LGM for both species. For *P. mantiqueirensis* the distribution during the LIG resembled that on the present but including areas with elevated occurrence probability further south. However, for *C. elegans* the distribution during this period was the smallest considering all models.

## Discussion

Here, we evaluated the genetic diversity based on plastid sequences and nuclear microsatellites [see Supporting Information—Table S10] of two narrowly distributed and rare species, *P. mantiqueirensis* and *C. elegans*, which represent the northern limit of these genera’s distribution. Each one of these species grows in disjunct habitats from the rest of species in their respective genera, in micro-environments located at extremes of elevation (Stehmann and Semir 2001; Stehmann *et al.* 2009), and, for *C. elegans*, a unique and particularly inhospitable soil type (Skirycz *et al.* 2014).

*Petunia* and *Calibrachoa* are two genera with recent diversification histories dating back to the Pleistocene (Lorenz-Lemke *et al.* 2010; Fregonezi *et al.* 2013; Särkinen *et al.* 2013). Both genera are distributed in South America and present widespread and narrowly distributed species, several of them being associated with particular habitats. Phylogeographic and phylogenetic studies of these two genera resulted in the proposal of two main patterns of distribution of genetic diversity and species diversification: (i) historical events, such as climate changes over glacial periods that affected the distribution of species driving allopatric speciation as proposed for *Petunia* species from the highlands (Lorenz-Lemke *et al.* 2010), and *C. heterophylla* (Mäder *et al.* 2013) and *P. integrifolia* (Ramos-Fregonezi *et al.* 2015) from the Atlantic Coastal Plain in south Brazil; and (ii) ecological factors, such as selective pressure of pollinators on morphological traits as indicated by the highly specialized interactions with their pollinators (Fregonezi *et al.* 2013; Sheehan *et al.* 2016; Rodrigues *et al.* 2018) or even climatic conditions (Barros *et al.* 2015). Both genera originated from an ancestor that inhabited the Pampas region at sea level and, secondarily, migrated to highlands, thereby colonizing the northern part of the genera’s distributions in south-eastern Brazil (Reck-Kortmann *et al.* 2015) during the Pleistocene glacial cycles as the open-field areas advanced (Behling 2002). In that time, during the cold and dry periods (glacial periods), species from open fields expanded to the north and, when climate turns warmer and more humid (interglacial periods), these populations were fragmented because forest growth and were restricted to the higher elevations (Lorenz-Lemke *et al.* 2010). Our demographic modelling of colonization for *C. elegans* and *P. mantiqueirensis* supports this.

In comparison with other *Petunia* species, *P. mantiqueirensis* has lower variability indices based on both plastid and nuclear markers than, for example, the also rare and endemic *P. secreta* (π = 0.17 % and nine cpDNA haplotypes; Turchetto *et al.* 2016) or the widespread *P. integrifolia* subsp*. depauperata* (π = 0.13 % and 25 haplotypes; Ramos-Fregonezi *et al.* 2015) and *P. axillaris* (π = 0.22 % and 35 haplotypes; Turchetto *et al.* 2014). Based on nuclear diversity, especially considering AR, other endemic species [*P. secreta*, AR = 5.91; and *P. exserta*, AR = 4.80 (Turchetto *et al.* 2016)] feature higher genetic variability than *P. mantiqueirensis* but are not significantly different from those that are widespread [*P. axillaris*, AR = 6.30 (Turchetto *et al.* 2015); *P. integrifolia* subsp. *depauperata*, AR = 4.30 (Silva-Arias *et al.* 2017)], suggesting that the reduced variability in *P. mantiqueirensis* is not a common characteristic of endemic species in the *Petunia* genus.

*Calibrachoa elegans* presented even lower genetic diversity indices compared to other *Calibrachoa* species as well as *P. mantiqueirensis*. The four micro-endemic highland species, *C. eglandulata*, *C. sendtneriana*, *C. serrulata* and *C. spathulata*, which have population sizes and geographical ranges similar to *C. elegans*, possess greater genetic diversity indices for both plastid and nuclear markers (John *et al.* 2019), whereas based on plastid diversity, the widespread species *C. sellowiana* (π = 0.088 %; J. N. Fregonezi *et al*., unpubl. data) and *C. heterophylla* (π = 0.41 %; Mäder *et al.* 2013) also have greater genetic diversity than *C. elegans.*

The diversification of highland species in *Petunia* and *Calibrachoa* has been discussed based on allopatric speciation as the result of isolation and fragmentation from a widespread ancient population during interglacial periods when forest species advanced on the open fields. The consecutive cycles of expansion and contraction of grasslands and forests, which changed the geographical distribution of the species, probably led to population fragmentation and possible local adaptation and population differentiation. More so than the north-east limit for the genera distribution, *P. mantiqueirensis* and *C. elegans* are the species that reached the highest elevation. Their restricted distribution probably corresponds to relics of a once widespread population that was fragmented by forest expansion during interglacial periods in the Pleistocene (Behling 2002), which suggests their requirement for a cold habitat and dispersion during cooler periods of the Pleistocene, compatible with previous findings for highlands petunias (Lorenz-Lemke *et al.* 2010). As commonly expected for rare and endemic plant species (Cole 2003), *P. mantiqueirensis* and *C. elegans* display low levels of genetic diversity, which is consistent with long periods as small and isolated populations.

The single haplotype found for *P. mantiqueirensis* is shared with *P. altiplana* and *P. bonjardinensis* (Lorenz-Lemke *et al.* 2010) and a very short genetic distance was observed between this and remained haplotypes found in other highlands species, reinforcing the recent diversification hypothesis for this group. Although low genetic diversity was found based on nuclear data, no indication of inbreeding was observed, which is consistent with the status of being self-incompatible and bee-pollinated proposed for *P. mantiqueirensis* (F. F. Araújo, UFMG, pers. comm.).

*Petunia mantiqueirensis* features a strict relationship with its bee pollinator (*Pseudagaspostemon fluminensis*) that forages pollen on plants returning at the same plot between 5 and 13 days after the first visit (F. F. Araújo, UFMG, pers. comm.). The maximum foraging distance for this genus of small and solitary bees is a maximum of 300 m (Gathmann and Tscharntke 2002; Zurbuchen *et al.* 2010). The distance among populations of *P. mantiqueirensis* ranges from ~0.9 to 14.2 km, and it is unlikely that this small bee promotes pollen flow between different patches, restricting it to an inside-patch flow. Thus, considering the absence of inbreeding and the improbability of gene flow among populations, a widely spread ancestor population that became recently fragmented is the most probable explanation for the observed genetic diversity of this species.

The reduced pollen flow among fragmented, isolated and discrete populations composed of relatively few individuals and the restricted seed dispersal are common characteristics of the majority of *Petunia* species (Stehmann *et al.* 2009) that can contribute to a depletion of overall genetic diversity over evolutionary time (Ellstrand and Elam 1993).

*Calibrachoa elegans* is an example of a rare species with habitat specificity, occurring only in four known populations. The severe environmental conditions (rigorous rainfall regime with 4 months of dry season) combined with the oscillations in the size of the species distribution areas based on the past modelling distribution may be a probable explanation for the lack of variation in *C. elegans*. Despite *C. elegans* being described as self-incompatible and bee-pollinated (Stehmann and Semir 2001), a significant deficit of heterozygotes and inbreeding values account for the distance among populations. Two main reasons can explain these results: mates preferentially occur between related individuals and/or self-incompatibility breaks. Self-incompatibility can be transitional in some populations dependent on environmental or biological conditions, like pollinator availability or even pollen sources (Nason and Hamrick 1997; Dobeš *et al.* 2017), or by fixation of mutations that result in non-self-incompatibility (Nasrallah *et al.* 2004). In addition, the positioning of reproductive organs in *C. elegans* is favourable to selfing.

We found stronger genetic differentiation among *C. elegans* populations than among populations of *P. mantiqueirensis*. *Calibrachoa elegans* is pollinated by *Hexantheda missionica* bees (Stehmann and Semir 2001), a species in which females collect pollen and nectar and effectively pollinate flowers, whereas males only use the flowers as a source of nectar, a place to land between patrol flights in search of females for mating, and as night shelter. This pollinator behaviour combined with the distance among plant patches can lead to low pollen flow and consequently strong differentiation and isolation.

Here, we add genetic information to several other criteria (CNCFlora) to confirm the endangered status for these two species, *P. mantiqueirensis* and *C. elegans*: their small and fragmented local populations, habitat specificity, restricted geographical range and low genetic diversity. Our results suggest a strong association between rarity and low genetic diversity related to the evolutionary history of these species. Species with a restricted distribution that are rare and with low diversity can be susceptible to depression because of the inbreeding and loss of natural genetic variability. Genetic drift tends to further diminish genetic diversity (Ellegren and Ellegren 2016; Thurman and Barrett 2016) resulting in allele fixation because of the low effective size. This phenomenon is more common on the edge of species distributions, where expansion and contraction are common (Eckert *et al.* 2008; Excoffier *et al.* 2009; Angert 2014; Hargreaves and Eckert 2014). Our findings are in accordance with this.

As in *P. secreta* (Turchetto *et al.* 2016), the main risk to *P. mantiqueirensis* and *C. elegans* is the fragmented habitat, population size and anthropization of their environment. These two species are adapted to specific habitats and changes in their environment can lead these species to extinction. They are currently threatened and their preservation *in situ* is necessary, especially because they are suffering from habitat loss. In an extreme situation, an *ex situ* intervention could be carried out, with the creation of a seed bank to re-introduce the plant to their natural environment. Through this contrasting pattern of rare species, which is associated with evolutionary history, it is important to produce detailed studies evaluating the genetic diversity of these species to create an efficient conservation plan for these two species that represent live relics from a past distribution of their respective genera.

## Sources of Funding

This work was supported by the Conselho Nacional de Desenvolvimento Científico e Tecnológico (CNPq), the Coordenação de Aperfeiçoamento de Pessoal de Nível Superior (CAPES), and the Programa de Pós Graduação em Genética e Biologia Molecular da Universidade Federal do Rio Grande do Sul (PPGBM-UFRGS). A.B. was recipient of National Institutes for Science and Technology (INCT) in Ecology, Evolution and Biodiversity Conservation scholarship. G.M. was supported by PNPD-CAPES/PPGBot-UFRGS and C.T. was supported by PNPD-CAPES/PPGBM-UFRGS.

## Contributions by the Authors

A.B. and L.B.F. planned, designed and led the project; A.B., G.M., J.N.F. conducted the experiments; A.B, C.T., A.L.A.S., G.M. and S.L.B. ran the analyses; A.B. and L.B.F. wrote most of the text; L.B.F. provided reagents and equipment to develop the experiments. All authors contributed in the preparation of the study and have commented on and approved the final manuscript.

## Supporting Information

The following additional information is available in the online version of this article—

**Table S1.** *Petunia mantiqueirensis* and *Calibrachoa elegans* collection points.

**Table S2.** Characteristics and PCR conditions of the microsatellite loci amplified for *Petunia mantiqueirensis* and *Calibrachoa elegans*.

**Table S3.** Prior values (minimum and maximum with uniform distribution) for the parameters employed for the four demographic scenarios (Fig. 2) with the DIYABC approach. Effective sizes are in number of individuals and times are in number of generations (generation time of 1 year).

**Table S4.** Additional information of approximate Bayesian computation (ABC) analyses.

**Table S5.** Geographic coordinates of *Petunia mantiqueirensis* and *Calibrachoa elegans* occurrence records used in the ENM.

**Table S6.** Characterization of 10 microsatellites analysed on *Petunia mantiqueirensis* collection sites.

**Table S7.** Pairwise *F*_ST_ values estimated for seven *Petunia mantiqueirensis* collection sites. (*) Significant values (*P* < 0.01).

**Table S8.** Characterization of five microsatellites analysed on *Calibrachoa elegans* collection sites.

**Table S9.** AICc scores and area under the receiver operating characteristic curve (AUC) values of the tested models.

**Table S10.** Data availability: Individual allele per microsatellite loci.

**Figure S1.** Clustering of individuals according to a discriminant analysis of principal components (DAPC) scatterplot performed based on SSR genotypes for: (A) seven populations of *Petunia mantiqueirensis* and (B) four populations of *Calibrachoa elegans*.

## Supplementary Material

plz037_suppl_Supplementary_Material

plz037_suppl_Supplementary_Table_S10
