# Supplementary material for: How diverse can rare species be on the margins of genera distribution?
Source: AoB Plants. 2019 Jul 9;11(4):plz037. doi: 10.1093/aobpla/plz037 (PMC6677564; doi:10.1093/aobpla/plz037)
Supplement: plz037_suppl_Supplementary_Material [file plz037_suppl_supplementary_material.pdf]

How diverse can rare species be on the margins of genera distribution?

Alice Backes, Geraldo Mäder, Caroline Turchetto, Ana Lucia Segatto, Jeferson Fregonezi, Sandro Bonatto, Loreta Freitas

Table S1. *Petunia mantiqueirensis* and *Calibrachoa elegans* collection points

Species	Site	N	Geographical coordinates	Voucher
Petunia mantiqueirensis	Mant1	13	22º39'32.00"S / 45º51'20.00"W	BHCB 74352
	Mant2	3	22º42'38.00"S / 45º55'54.00"W	BHCB 56585
	Mant3	14	22º42'35.00"S / 45º56'50.00"W	BHCB 78265
	Mant4	2	22º40'57.00"S / 45º52'49.00"W	BHCB 78268
	Mant5	4	22º40'31.00"S / 45º52'33.00"W	BHCB 78269
	Mant6	1	22°41'00.00"S / 45°55'00.00"W	JRS 3087
	Mant7	1	22º46'36.00"S / 45º54'32.00"W	NA
Calibrachoa elegans	Eleg1	16	20º05'35.00"S / 43º59'01.00"W	BHCB 41024
	Eleg2	21	21º36'39.41"S / 44º07'34.33"W	BHCB 103115
	Eleg3	15	20º06'11.17"S / 43º56'46.33"W	BHCB 104337
	Eleg4	29	20º06'09.83"S / 43º59'15.78"W	BHCB 71641

N – number of individuals per site; BHCB – Herbarium at Universidade Federal de Minas Gerais, Belo Horizonte, MG, Brazil; JRS – collector number for João Renato Stehmann (available at BHCB, UFMG, Brazil); NA – not available

Table S2. Characteristics and PCR conditions of the microsatellite loci amplified for *Petunia mantiqueirensis* and *Calibrachoa elegans*

Species	Locus	Chr	SR	Repeat motif	T_a (°C)	C	DYE
Petunia mantiqueirensis	PM 8	IV	149-165	$(AAGA)_{10}$	51	32x	FAM
	PM 167	V	287-308	$(TTC)_{12}$	51,5	32x	HEX
	PM 101	I	265-271	$(CTT)_6$	51	32x	HEX
	PM 117	VI	289-298	$(GTG)_7$	51	35x	HEX
	PM 177	V	188-202	$(CA)_{11}$	54	35x	HEX
	PM 21	II	129-132	$(TCA)_8$	51	32x	NED
	PM 184	VII	84-102	$(GAA)_6$	54	34x	NED
	PM 191	III	171-201	$(TTG)_6$	51	32x	HEX
	PM 173	IV	142-175	$(GCA)_6$	52	32x	FAM
	PM 63	IV	334-340	$(ACAGCA)_5$	52	32x	FAM
Calibrachoa elegans	CHE 33	NA	185-215	$(GA)_{20}$	50	35x	PET
	CHE 34	NA	227-241	$(TC)_{10}$	50	35x	FAM
	CHE 59	NA	100-112	$(CT)_7$	54	35x	FAM
	CHE 85	NA	306-336	$(TG)_6(GA)_8$	49	35x	VIC
	CHE 126	NA	312-348	$(GA)_{16}$	48	35x	NED

Chr - microsatellite location (chromosome number); SR - size range; T_a - annealing temperatures; C - number of cycles of PCR; NA - Not available.

Table S3. Prior values (minimum and maximum with uniform distribution) for the parameters employed for the four demographic scenarios (Fig. 2) with the DIYABC approach. Effective sizes are in number of individuals and times are in number of generations (generation time of one year)

Scenario/Parameters	Minimum	Maximum
Scenario 1		
Ne	10	500,000
Scenario 2		
Ne2	10	15,000
*Na2**	10,000	500,000
t	10	50,000
Scenario 3		
Ne3	10	500,000
*Na3**	10	500,000
t	10	50,000
Scenario 4		
Ne4	10	500,000
Nb	10	100,000
Na4	1,000	500,000
*t1**	4,000	12,000
*t2**	17,000	50,000

* *Ne2<Na2; Na3<Ne3; Nb4<Ne4; Ne4>Nb4*

Table S4. Additional information of ABC analyses

P. mantiqueirensis

POSTERIOR PROBABILITIES OF SCENARIOS

Candidate scenarios : [1, 2, 3, 4]
Number of simulated data sets : 8000000

Direct approach

closest	scenario 1	scenario 2	scenario 3	scenario 4
50	0.0000 [0.0000,0.0000]	1.0000 [1.0000,1.0000]	0.0000 [0.0000,0.0000]	0.0000 [0.0000,0.0000]
100	0.0000 [0.0000,0.0000]	1.0000 [1.0000,1.0000]	0.0000 [0.0000,0.0000]	0.0000 [0.0000,0.0000]
150	0.0000 [0.0000,0.0000]	1.0000 [1.0000,1.0000]	0.0000 [0.0000,0.0000]	0.0000 [0.0000,0.0000]
200	0.0000 [0.0000,0.0000]	1.0000 [1.0000,1.0000]	0.0000 [0.0000,0.0000]	0.0000 [0.0000,0.0000]
250	0.0000 [0.0000,0.0000]	1.0000 [1.0000,1.0000]	0.0000 [0.0000,0.0000]	0.0000 [0.0000,0.0000]
300	0.0000 [0.0000,0.0000]	1.0000 [1.0000,1.0000]	0.0000 [0.0000,0.0000]	0.0000 [0.0000,0.0000]
350	0.0000 [0.0000,0.0000]	1.0000 [1.0000,1.0000]	0.0000 [0.0000,0.0000]	0.0000 [0.0000,0.0000]
400	0.0000 [0.0000,0.0000]	1.0000 [1.0000,1.0000]	0.0000 [0.0000,0.0000]	0.0000 [0.0000,0.0000]
450	0.0000 [0.0000,0.0000]	1.0000 [1.0000,1.0000]	0.0000 [0.0000,0.0000]	0.0000 [0.0000,0.0000]
500	0.0000 [0.0000,0.0000]	1.0000 [1.0000,1.0000]	0.0000 [0.0000,0.0000]	0.0000 [0.0000,0.0000]

Logistic approach

n	scenario 1	scenario 2	scenario 3	scenario 4
8000	0.0029 [0.0002,0.0056]	0.9971 [0.9944,0.9998]	0.0000 [0.0000,0.0000]	0.0000 [0.0000,0.0000]
16000	0.0050 [0.0020,0.0081]	0.9950 [0.9919,0.9980]	0.0000 [0.0000,0.0000]	0.0000 [0.0000,0.0000]
24000	0.0057 [0.0032,0.0082]	0.9943 [0.9918,0.9968]	0.0000 [0.0000,0.0000]	0.0000 [0.0000,0.0000]
32000	0.0064 [0.0040,0.0089]	0.9936 [0.9911,0.9960]	077.0000 [0.0000,0.0000]	0.0000 [0.0000,0.0000]
40000	0.0073 [0.0049,0.0097]	0.9927 [0.9903,0.9951]	0.0000 [0.0000,0.0000]	0.0000 [0.0000,0.0000]

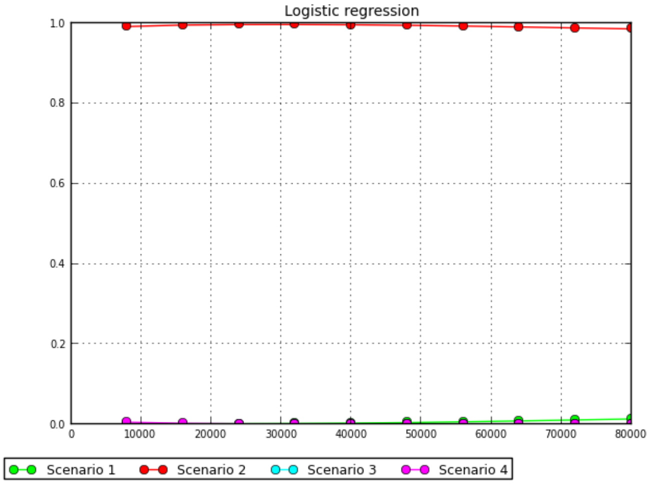

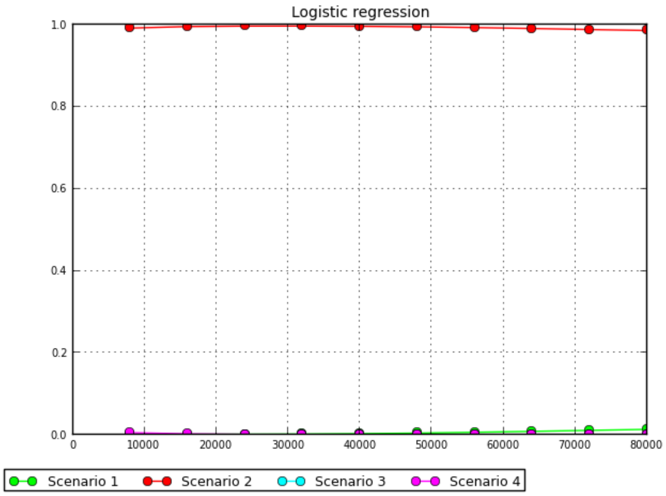

POSTERIOR DISTRIBUTION OF PARAMETER
Transformation LOGIT of parameters
Chosen scenario(s) : 2
Number of simulated data sets : 2000000
Number of selected data sets : 20000

Parameter	mean	median	mode	q050	q950
Ne2	7.43e+002	6.80e+002	5.34e+002	3.53e+002	1.33e+003
t	7.09e+003	4.13e+003	2.00e+003	9.01e+002	2.62e+004
Na2	2.37e+005	2.28e+005	7.72e+004	3.32e+004	4.68e+005

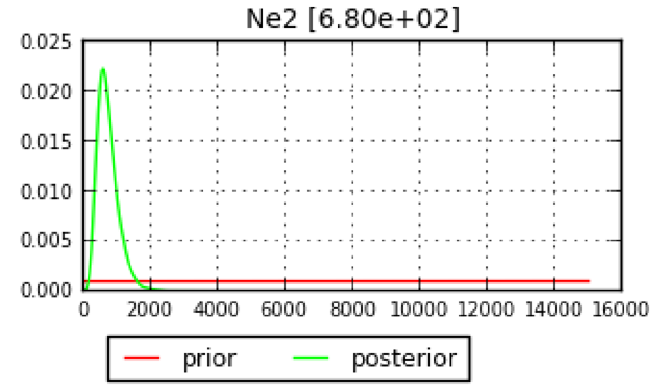

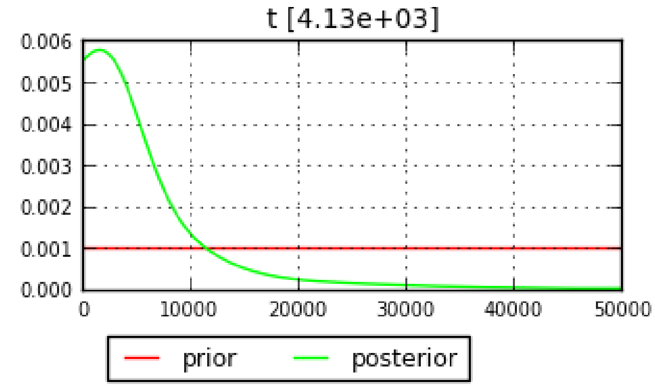

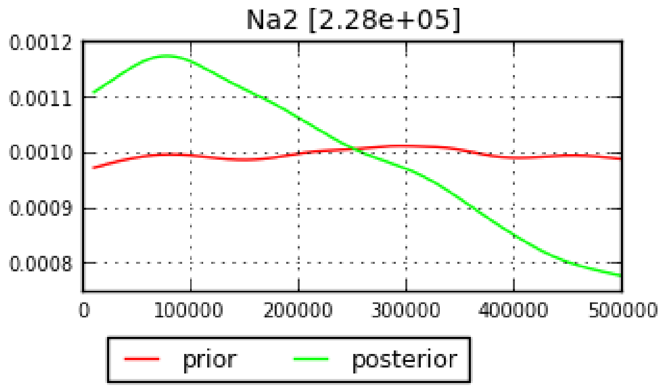

CONFIDENCE IN SCENARIO CHOICE

Number of simulated data sets : 8000000
Computation of posterior sample using plain summary statistics
Sample size of the posterior distribution (=simulated datasets closest to observed) : 500
Direct approach : number of selected data sets : 500
Logistic regression : number of selected data sets : 80000
Candidate scenarios : 1, 2, 3, 4
Results obtained with plain summary statistics
Posterior predictive error (computed over 1000 data sets):
Direct approach : 0.000
Logistic approach : 0.000

POSTERIOR MODEL CHECKING

Chosen scenario : 2
Number of simulated data sets used to compute posterior : 2000000
Number of simulated data sets used in the local regression : 20000
Number of data sets simulated from the posterior : 1000
Transformation of parameters : Logit

summary statistics	observed value	proportion (simulated<observed)
NAL_1_1	3.9000	0.4050
HET_1_1	0.4356	0.1405
VAR_1_1	1.9501	0.4790
MGW_1_1	0.6290	0.2850

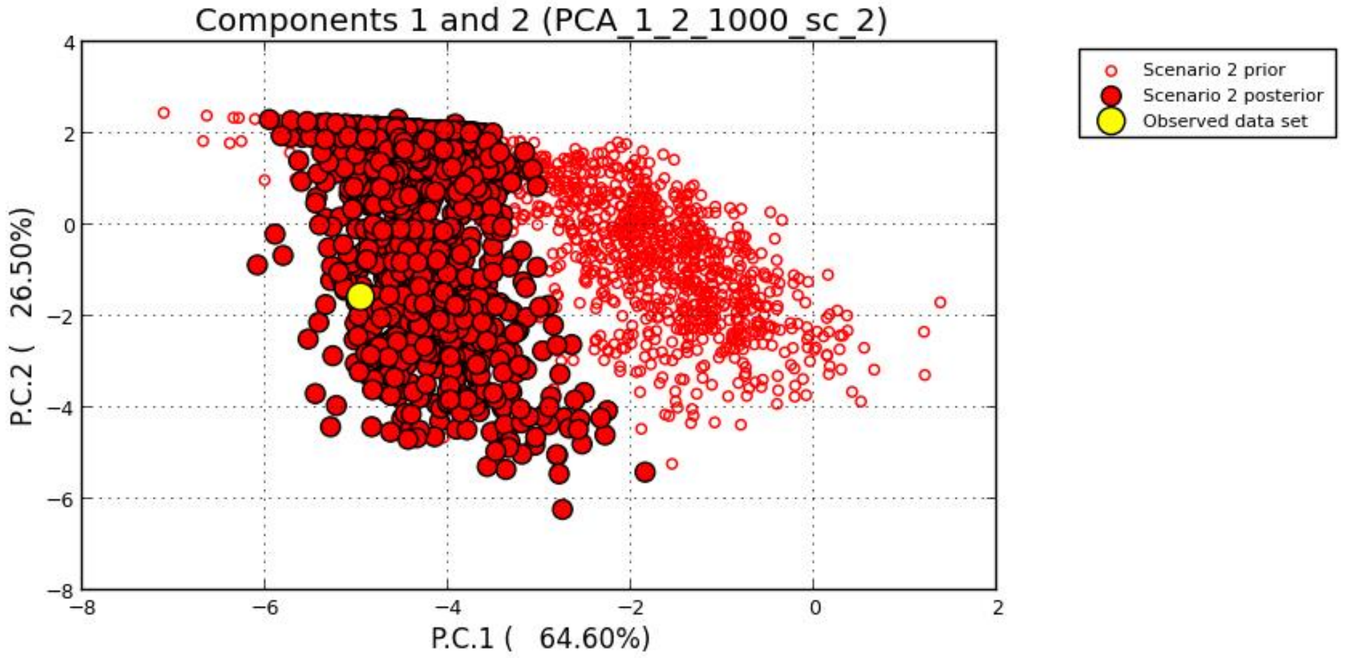

BIAS AND MEAN SQUARE ERROR ANALYSIS

Transformation LOGIT of parameters
Chosen scenario : 2
Number of simulated data sets : 2000000
Number of selected data sets : 20000
Results based on 500 test data sets

			Averages		
Parameter	True values	Means	Medians	Modes	
Ne2	8.90E+02	1.10E+03	1.01E+03	8.59E+02	
t	6.72E+03	1.73E+04	1.63E+04	1.51E+04	
Na2	2.57E+05	2.68E+05	2.71E+05	2.93E+05	
µmic_1	6.72E-04	7.05E-04	6.85E-04	5.11E-04	
		Mean Relative Bias			
Parameter	Means	Medians	Modes		
Ne2	0.284	0.1835	0.0049		
t	2.362	2.0754	1.6093		
Na2	1.076	1.0902	1.1686		
µmic_1	0.087	0.0565	-0.2122		
			Square root of mean square error/true value		
Parameter	RRMISE	RMeanAD	Mean	Median	Mode
Ne2	0.784	0.526	0.545	-9.713	0.468
t	4.143	2.482	3.412	-6.005	3.229
Na2	3.655	1.511	3.104	-3.064	3.108
µmic_1	0.311	0.242	0.22	-0.266	0.205

C. elegans

POSTERIOR PROBABILITIES OF SCENARIOS

Candidate scenarios : [1, 2, 3, 4]
Number of simulated data sets : 8000000
Summary statistics have been replaced by LDA component
Direct approach

closest	scenario 1	scenario 2	scenario 3	scenario 4
50	0.0000 [0.0000,0.0000]	0.9400 [0.7318,1.0000]	0.0200 [0.0000,0.1427]	0.0400 [0.0000,0.2118]
100	0.0100 [0.0000,0.0972]	0.9200 [0.6822,1.0000]	0.0100 [0.0000,0.0972]	0.0600 [0.0000,0.2682]
150	0.0067 [0.0000,0.0780]	0.9067 [0.6517,1.0000]	0.0067 [0.0000,0.0780]	0.0800 [0.0000,0.3178]
200	0.0050 [0.0000,0.0668]	0.9150 [0.6705,1.0000]	0.0050 [0.0000,0.0668]	0.0750 [0.0000,0.3059]
250	0.0080 [0.0000,0.0861]	0.9200 [0.6822,1.0000]	0.0040 [0.0000,0.0593]	0.0680 [0.0000,0.2887]
300	0.0100 [0.0000,0.0972]	0.9300 [0.7064,1.0000]	0.0033 [0.0000,0.0539]	0.0567 [0.0000,0.2593]
350	0.0086 [0.0000,0.0894]	0.9286 [0.7028,1.0000]	0.0029 [0.0000,0.0496]	0.0600 [0.0000,0.2682]
400	0.0075 [0.0000,0.0831]	0.9275 [0.7002,1.0000]	0.0025 [0.0000,0.0463]	0.0625 [0.0000,0.2747]
450	0.0067 [0.0000,0.0780]	0.9289 [0.7036,1.0000]	0.0022 [0.0000,0.0435]	0.0622 [0.0000,0.2740]
500	0.0060 [0.0000,0.0737]	0.9300 [0.7064,1.0000]	0.0020 [0.0000,0.0412]	0.0620 [0.0000,0.2734]

Logistic approach

n	scenario 1	scenario 2	scenario 3	scenario 4
8000	0.0024 [0.0010,0.0038]	0.9916 [0.9895,0.9938]	0.0001 [0.0000,0.0001]	0.0059 [0.0043,0.0076]
16000	0.0018 [0.0010,0.0025]	0.9953 [0.9943,0.9963]	0.0000 [0.0000,0.0001]	0.0029 [0.0023,0.0035]
24000	0.0019 [0.0013,0.0026]	0.9965 [0.9957,0.9972]	0.0000 [0.0000,0.0000]	0.0016 [0.0013,0.0019]
32000	0.0024 [0.0017,0.0031]	0.9966 [0.9959,0.9974]	0.0000 [0.0000,0.0000]	0.0009 [0.0008,0.0011]
40000	0.0034 [0.0025,0.0043]	0.9960 [0.9951,0.9969]	0.0000 [0.0000,0.0000]	0.0006 [0.0005,0.0007]
48000	0.0048 [0.0037,0.0060]	0.9947 [0.9936,0.9958]	0.0000 [0.0000,0.0000]	0.0005 [0.0004,0.0005]
56000	0.0068 [0.0055,0.0082]	0.9928 [0.9914,0.9941]	0.0000 [0.0000,0.0000]	0.0004 [0.0003,0.0005]
64000	0.0091 [0.0074,0.0107]	0.9906 [0.9889,0.9922]	0.0000 [0.0000,0.0000]	0.0004 [0.0003,0.0004]
72000	0.0115 [0.0097,0.0133]	0.9881 [0.9863,0.9899]	0.0000 [0.0000,0.0001]	0.0004 [0.0003,0.0004]
80000	0.0139 [0.0119,0.0158]	0.9857 [0.9837,0.9877]	0.0000 [0.0000,0.0001]	0.0004 [0.0003,0.0005]

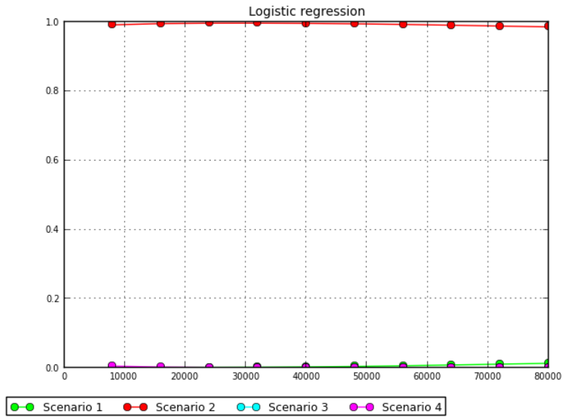

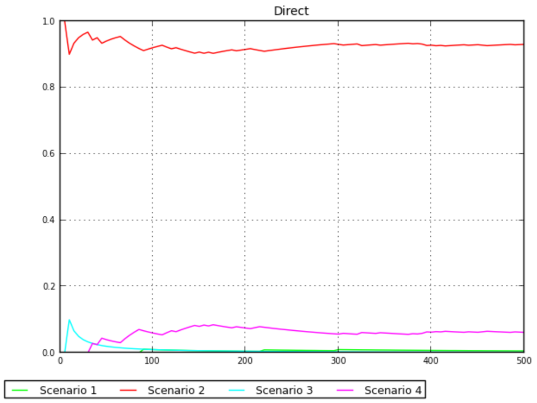

POSTERIOR DISTRIBUTION OF PARAMETER

Transformation LOGIT of parameters
Chosen scenario(s) : 2
Number of simulated data sets : 2000000
Number of selected data sets : 20000

Parameter	mean	median	mode	q050	q950
**Ne2**	**7.35e+003**	**6.91e+003**	**4.88e+003**	**3.18e+003**	**1.30e+004**
**t**	**2.79e+004**	**2.75e+004**	**2.52e+004**	**9.03e+003**	**4.74e+004**
**Na2**	**2.81e+005**	**2.97e+005**	**4.77e+005**	**3.67e+004**	**4.83e+005**

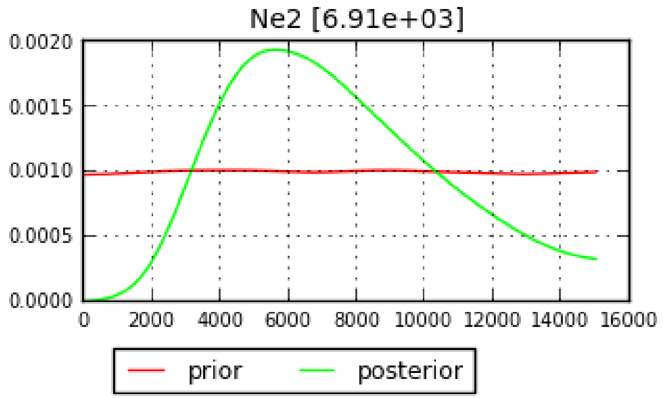

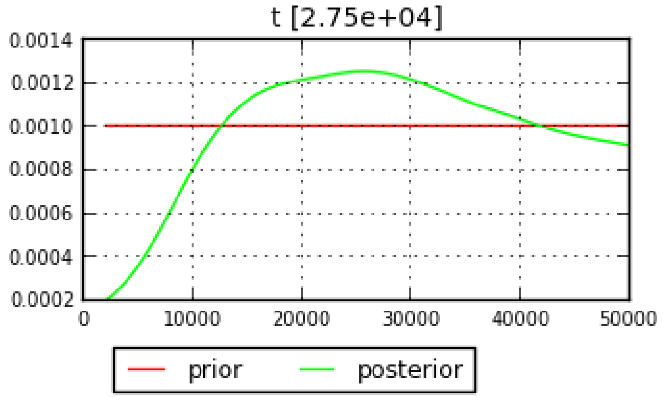

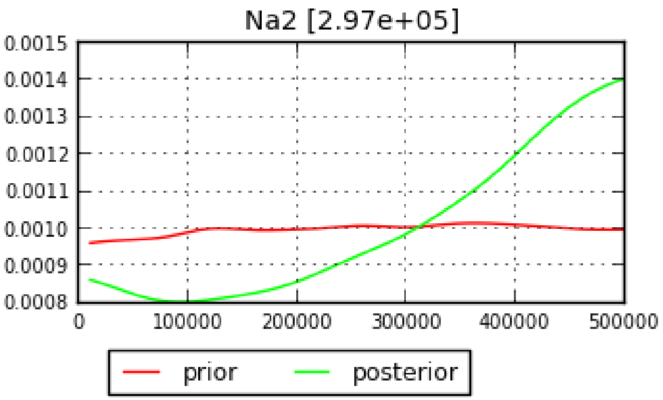

CONFIDENCE IN SCENARIO CHOICE

Number of simulated data sets : 1000000
Computation of posterior sample using plain summary statistics
Sample size of the posterior distribution (=simulated datasets closest to observed) : 500
Direct approach : number of selected data sets : 500
Logistic regression : number of selected data sets : 10000
Candidate scenarios : 1, 2, 3, 4
Summary statistics have been replaced by components of a Linear Discriminant Analysis
Posterior predictive error (computed over 1000 data sets):
Direct approach : 0.206
Logistic approach : 0.161

POSTERIOR MODEL CHECKING

Chosen scenario : 2
Number of simulated data sets used to compute posterior : 2001873
Number of simulated data sets used in the local regression : 20018
Number of data sets simulated from the posterior : 1000
Transformation of parameters : Logit
summary observed proportion
statistics value (simulated<observed)

```
NAL_1_1 7.8000 0.4580
HET_1_1 0.6755 0.1810
VAR_1_1 4.2402 0.2690
MGW_1_1 1.0986 0.9990 (***)
```

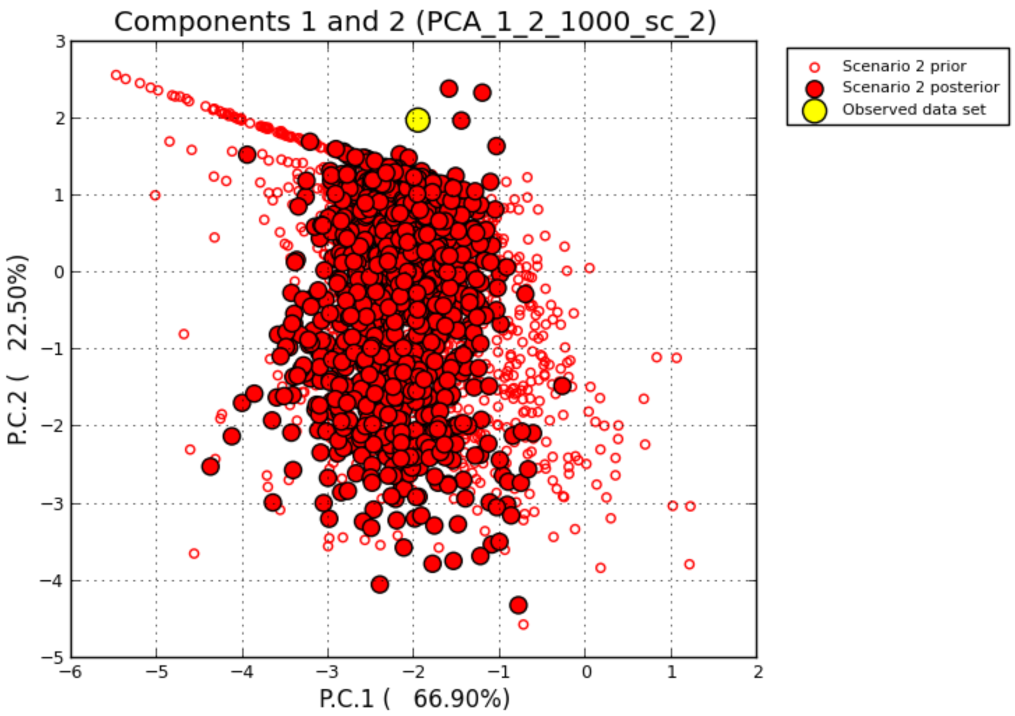

BIAS AND MEAN SQUARE ERROR ANALYSIS

Transformation LOGIT of parameters
Chosen scenario : 2
Number of simulated data sets : 2001873
Number of selected data sets : 20018
Results based on 500 test data sets

			Averages		
Parameter	True values	Means	Medians	Modes	
Ne2	7.340e+003	7.029e+003	6.551e+003	4.920e+003	
t	2.881e+004	2.760e+004	2.761e+004	3.053e+004	
Na2	2.745e+005	2.542e+005	2.496e+005	1.449e+005	
µmic_1	4.234e-004	4.979e-004	4.664e-004	3.422e-004	
		Mean Relative Bias			
Parameter	Means	Medians	Modes		
Ne2	0.125	0.0457	-0.2203		
t	0.222	0.2027	0.2514		
Na2	0.737	0.6921	-0.3408		
µmic_1	0.531	0.4093	-0.0583		
			Square root of mean square error/true value		
Parameter	RRMISE	RMeanAD	Mean	Median	Mode
Ne2	0.806	0.579	0.573	0.555	0.546
t	1.091	0.638	0.856	0.846	1.036
Na2	3.068	1.253	2.505	2.413	1.036
µmic_1	1.327	0.836	0.995	0.885	0.654

Table S5. Geographic coordinates of the *Petunia mantiqueirensis* and *Calibrachoa elegans* occurrence records used in Ecological Niche Modelling

Species	Geographical coordinates
Petunia mantiqueirensis	23°09′29.99″S 46°31′03.00″W
	23°09′14.00″S 46°29′10.00″W
	23°07′52.00″S 46°27′47.99″W
	22°48′29.16″S 46°04′45.84″W
	22°46′36.00″S 45°54′32.00″W
	22°45′19.08″S 46°08′40.92″W
	22°45′06.00″S 45°55′55.99″W
	22°42′38.00″S 45°55′54.00″W
	22°42′35.00″S 45°56′50.00″W
	22°41′12.99″S 45°58′14.99″W
	22°40′60.00″S 45°55′00.00″W
	22°40′57.00″S 45°52′49.00″W
	22°39′32.04″S 45°51′20.88″W
Calibrachoa elegans	21°36′39.41″S 44°07′34.33″W
	20°29′58.92″S 43°51′28.08″W
	20°19′59.16″S 44°03′10.08″W
	20°17′15.00″S 43°30′29.15″W
	20°15′11.88″S 43°48′05.04″W
	20°08′35.88″S 44°11′58.92″W
	20°06′11.17″S 43°56′46.33″W
	20°06′09.83″S 43°59′15.78″W
	20°05′35.00″S 43°59′01.00″W
	19°59′08.16″S 43°50′48.12″W
	19°55′14.88″S 43°56′16.08″W

Table S6. Characterization of the 10 microsatellites per locus and per collection site of *Petunia mantiqueirensis*

Site/loci		PM8	PM167	PM101	PM117	PM177	PM21	PM184	PM191	PM173	PM63	Average
Mant1	N	3	4	3	2	2	1	5	1	4	2	-
	E	0	1	1	0	0	0	1	0	0	0	-
	H_E	0.465	0.406	0.283	0.517	0.323	-	0.732	-	0.721	0.489	0.492
	H_O	0.308	0.308	0.000*	0.615	0.077*	-	1.000*	-	1.000	0.750	0.579
	F_{IS}	0.347	0.250	1.000	-0.200	0.769	-	-0.387	-	-0.417	-0.571	-0.033
Mant2	N	3	3	1	2	2	2	3	1	2	2	-
	E	0	0	0	0	0	0	0	0	0	0	-
	H_E	0.600	0.733	-	0.533	0.333	0.500	0.733	-	1.000	0.500	0.616
	H_O	0.667	1.000	-	0.667	0.333	0.500	0.667	-	1.000	0.500	0.667
	F_{IS}	-0.143	-0.500	-	-0.333	0.000	0.000	0.111	-	-	0.000	-0.130
Mant3	N	4	4	2	3	2	2	2	3	6	2	-
	E	0	0	0	0	0	0	0	2	1	0	-
	H_E	0.513	0.729	0.071	0.690	0.137	0.349	0.476	0.163	0.847	0.431	0.441
	H_O	0.571	0.923	0.071	0.928	0.143	0.428	0.714	0.083*	0.800	0.583	0.524
	F_{IS}	-0.118	-0.280	0.000	-0.363	-0.040	-0.238	-0.529	0.500	0.059	-0.375	-0.199
Mant4	N	1	1	2	1	1	1	2	1	2	2	-
	E	0	0	0	0	0	0	0	0	0	0	-
	H_E	-	-	0.667	-	-	-	0.667	-	0.667	0.500	0.625
	H_O	-	-	1.000	-	-	-	1.000	-	1.000	0.500	0.875
	F_{IS}	-	-	-1.000	-	-	-	-1.000	-	-1.000	0.000	-0.750
Mant5	N	1	5	2	2	2	1	3	1	4	2	-
	E	0	1	0	0	0	0	0	0	0	0	-
	H_E	-	0.786	0.533	0.571	0.250	-	0.714	-	0.867	0.600	0.617
	H_O	-	0.750	0.667	0.500	0.250	-	0.500	-	1.000	1.000	0.667
	F_{IS}	-	0.053	-0.333	0.143	0.000	-	0.333	-	-0.200	-1.000	-0.109
Mant6	N	1	2	1	1	2	1	1	1	2	2	-
	E	0	0	0	0	0	0	0	0	1	0	-
	H_E	-	0.667	-	-	0.667	-	-	-	0.667	0.667	0.667
	H_O	-	1.000	-	-	1.000	-	-	-	1.000	1.000	1.000
	F_{IS}	-	-1.000	-	-	-1.000	-	-	-	-1.000	-1.000	-1.000
Mant7	N	2	2	1	1	1	2	2	1	NA	2	-
	E	0	0	0	0	0	0	0	0	0	0	-
	H_E	0.667	0.667	-	-	-	0.667	0.667	-	-	0.667	0.667
	H_O	1.000	1.000	-	-	-	1.000	1.000	-	-	1.000	1.000
	F_{IS}	-1.000	-1.000	-	-	-	-1.000	-1.000	-	-	-1.000	-1.000

N - number of alleles; E - number of private alleles; H_E - expected heterozygosity; H_O - observed heterozygosity; F_{IS} - inbreeding coefficient. * - significant Hardy-Weinberg equilibrium deviation after Bonferroni correction at $P = 0.05$; - not estimated

Table S7. Pairwise F_{ST} values estimated for the seven *Petunia mantiqueirensis* collection sites. (*) Significant values ($P < 0.01$)

Sites	Mant1	Mant2	Mant3	Mant4	Mant5	Mant6
Mant1	-					
Mant2	0.02	-				
Mant3	0.07*	-0.02	-			
Mant4	0.25*	0.21	0.23*	-		
Mant5	0.12	0.13	0.11*	0.13	-	
Mant6	0.19	0.16	0.25*	0.62	0.34	-
Mant7	0.10	0.16	0.08	0.49	0.16	0.49

Table S8. Characterization of the five microsatellites per locus and per collection site of *Calibrachoa elegans*

Site/ Loci		CHE33	CHE34	CHE59	CHE85	CHE126	Average
Eleg1	N	9	5	3	5	5	-
	E	2	1	0	0	0	-
	AR	9.000	4.876	2.966	4.637	4.692	5.234
	H_E	0.884	0.615	0.559	0.570	0.635	0.653
	H_O	0.750	0.687	0.600	0.625	0.437*	0.620
	F_{IS}	0.157	-0.122	-0.077	-0.099	0.318	0.052
Eleg2	N	3	2	2	1	2	-
	E	0	0	0	1	1	-
	AR	2.846	1.999	1.667	1.000	1.846	1.872
	H_E	0.528	0.284	0.055	-	0.097	0.241
	H_O	0.100*	0.048*	0.055	-	0.000*	0.051
	F_{IS}	0.815*	0.836	0.000	-	1.000	0.794
Eleg3	N	8	4	3	8	5	-
	E	3	1	0	4	0	-
	AR	7.760	3.920	3.000	7.292	4.997	5.394
	H_E	0.732	0.495	0.645	0.634	0.757	0.653
	H_O	0.615	0.308	0.428	0.733	0.385*	0.494
	F_{IS}	0.165	0.389	0.345	-0.162	0.502*	0.251
Eleg4	N	5	4	4	5	4	-
	E	0	0	1	0	0	-
	AR	4.792	3.951	3.304	4.187	3.950	4.037
	H_E	0.747	0.585	0.584	0.606	0.665	0.630
	H_O	0.552*	0.518*	0.552	0.621	0.273*	0.503
	F_{IS}	0.265	0.115	0.056	-0.024	0.596*	0.214

N - number of alleles; E - number of private alleles; AR - allele richness; H_E - expected heterozygosity; H_O - observed heterozygosity; F_{IS} - inbreeding coefficient. * - significant Hardy-Weinberg equilibrium deviation after Bonferroni correction at $P = 0.05$; - not estimated.

Table S9. AICc scores and AUC values of the tested models

	Calibrachoa elegans			*Petunia mantiqueirensis*		
Feature classe/regularization multiplier	Parameters	AICc scores	AUC	Parameters	AICc scores	AUC
Linear/1	6	294.098103615972	0.991	4	301.941219549238	0.997
Linear/2	5	290.258583486892	0.990	2	304.169948895271	0.997
Linear/3	3	283.904686119297	0.989	2	314.16795003051	0.997
Linear/4	3	289.037323135611	0.989	2	323.996535165391	0.997
Linear/5	3	293.878876028312	0.989	2	327.583170002366	0.997
Hinge/1	12	x	0.996	17	x	0.998
Hinge/2	12	x	0.996	18	x	0.997
Hinge/3	14	x	0.996	14	x	0.996
Hinge/4	8	368.050506467146	0.995	10	446.164446788444	0.995
Hinge/5	7	350.69302042209	0.994	14	x	0.995
Linear and quadratic/1	5	265.057365930087	0.994	4	284.268679954277	0.998
Linear and quadratic/2	5	280.173734188484	0.992	4	293.623775859498	0.998
Linear and quadratic/3	3	275.197659685732	0.991	4	301.994225704549	0.998
Linear and quadratic/4	3	279.409456640538	0.990	3	305.131326453217	0.997
Linear and quadratic/5	3	283.524376382974	0.990	3	312.069986625628	0.997
Linear, quadratic and hinge/1	5	257.803227037084	0.996	9	346.162688582082	0.998
Linear, quadratic and hinge/2	5	267.021425779002	0.996	7	316.390760375309	0.998
Linear, quadratic and hinge/3	5	276.825790389693	0.995	4	301.995036824683	0.998
Linear, quadratic and hinge/4	5	286.300823181957	0.994	3	305.130555975087	0.997
Linear, quadratic and hinge/5	4	287.670444222356	0.991	3	312.064009917421	0.997
Default/1	5	265.057365930087	0.994	4	272.568351591463	0.998
Default/2	5	280.173734188484	0.992	4	278.430896806343	0.998
Default/3	3	275.197659685732	0.991	3	278.88927298667	0.997
Default/4	3	279.409456640538	0.990	3	282.794896648943	0.997
Default/5	3	294.098103615972	0.990	3	286.412817472034	0.997

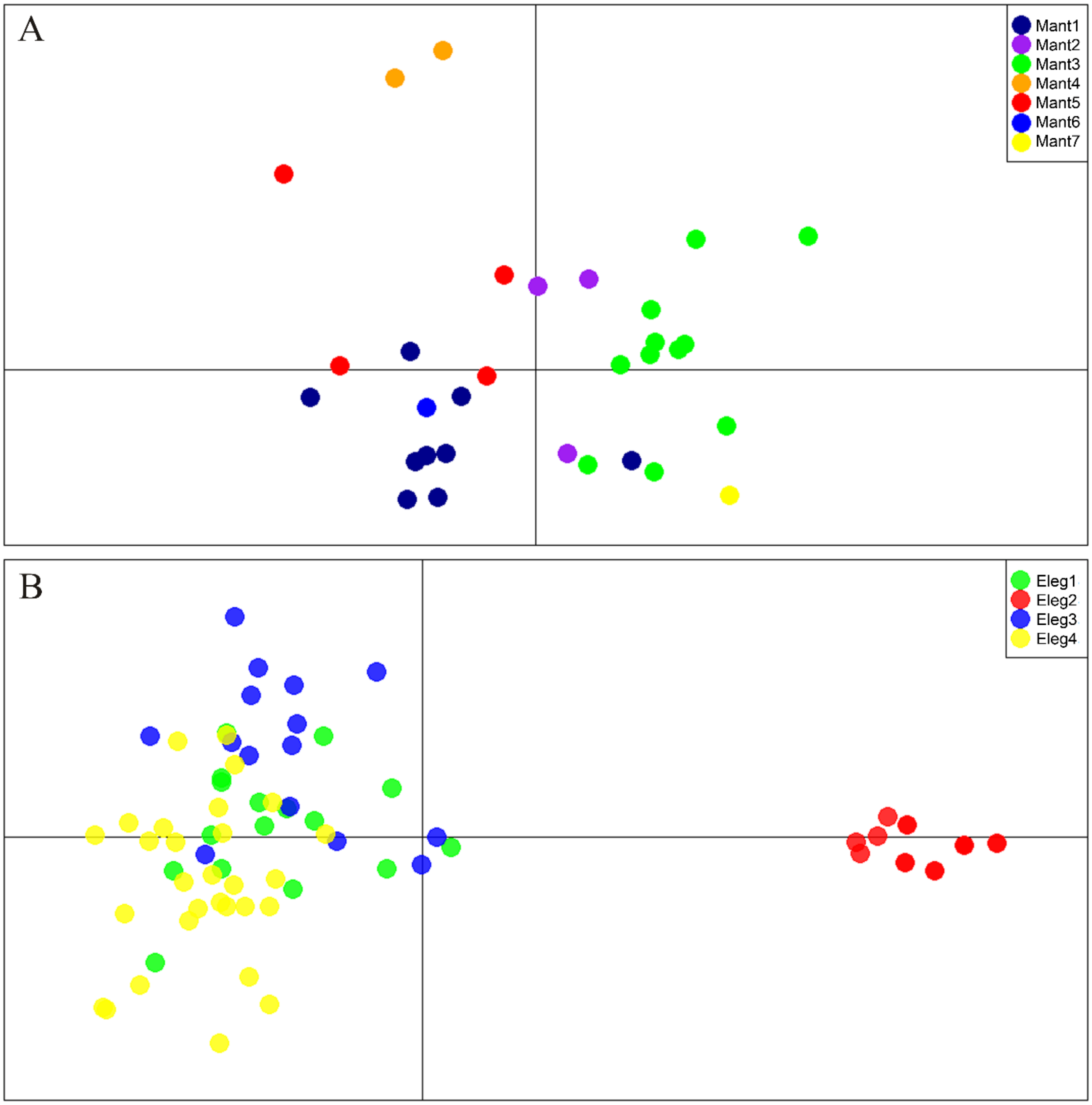

Figure S1. Clustering of individuals according to a DAPC scatterplot performed based on SSR genotypes for: (A) seven populations of *Petunia mantiqueirensis* and (B) four populations of *Calibrachoa elegans*.
